# Exploring glyco-signatures and their clinical implications: a special issue focused on glycosylation studies

**DOI:** 10.3724/abbs.2024143

**Published:** 2024-08-15

**Authors:** Haojia Lu, Xing Chen

**Affiliations:** 1 Institutes of Biomedical Sciences and Liver Cancer Institute Zhongshan Hospital Fudan University Shanghai 200032 China; 2 Department of Chemistry and NHC Key Laboratory of Glycoconjugates Research Fudan University Shanghai 200433 China; 3 College of Chemistry and Molecular Engineering Peking University Beijing 100871 China; 4 Beijing National Laboratory for Molecular Sciences Peking University Beijing 100871 China; 5 Peking-Tsinghua Center for Life Sciences Peking University Beijing 100871 China; 6 Synthetic and Functional Biomolecules Center Peking University Beijing 100871 China; 7 Key Laboratory of Bioorganic Chemistry and Molecular Engineering of Ministry of Education Peking University Beijing 100871 China

Glycosylation is one of the most common post-translational modifications, playing a crucial role in various physiological and pathological processes. An increasing number of studies suggest that research on glycosylation holds promise for the development of clinical diagnostic biomarkers and therapeutic targets. This special issue combines four research studies and eight reviews that cover a vast range of topics within glycosylation research field, contributed by specialists in the field from various regions and countries.

Considering the extensive correlation between glycosylation and various physiological and pathological processes, as well as the critical role of key technologies in glycosylation research, this special issue explores the role of glycosylation in cancer, infectious diseases, aging and associated disorders. Both N-linked and O-linked glycosylation, the two main forms of glycosylation modifications, were included. Additionally, the issue also reviews some major glycomics technologies used in glycosylation studies, including lectin microarrays, glycomics, and glycoproteomics, along with their applications in the research and development of glycan biomarkers and targets. Each topic focuses on different aspects of glycosylation, offering new insights and potential therapeutic strategies.

In the research study series, four topics are studied related to cancer and infectious diseases. An article by Cao *et al*. [1] systematically characterize anti-PD-1/PD-L1 immunotherapy-related changes in serum glycoproteins, revealing significant alterations in glycopeptide levels that correlate with treatment responses. This research suggests the potential of glycosylation profiling in guiding immunotherapy decisions and improving clinical outcomes for lung cancer patients. An article by Jiang *et al*. [2] focus on investigating the role of O-GlcNAcylation and O-GalNAc glycosylation in bladder cancer. They demonstrate how these modifications stabilize key glycosyltransferases and influence cancer cell behavior. This study offers insights into the interplay between different glycosylation pathways in cancer and potential therapeutic targets. Additionally, the role of glycosylation in viral and bacterial infections is illuminated in the studies by Xu *et al*. [3] and Zhang *et al*. [4], respectively. In the context of viral infection, the COVID-19 pandemic has driven extensive research into the molecular mechanisms of SARS-CoV-2. Xu *et al*. [3] investigate the O-glycosylation of the SARS-CoV-2 spike protein by host O-glycosyltransferases, identifying 15 O-glycosites and 10 distinct O-glycan structures. Their findings suggest that O-glycosylation stabilizes the trimeric spike protein structure, providing valuable insights into viral infection mechanisms and potential therapeutic targets. Regarding bacterial infection, tuberculosis remains one of the leading causes of death worldwide, and the efficacy of the Bacillus Calmette-Guérin (BCG) vaccine is variable. Zhang *et al*. [4] elucidate the role of macrophage mannose receptor (MR) in the antimycobacterial immune response during BCG vaccination. They demonstrate that MR deficiency impairs antigen presentation and immune response, indicating MR’s importance in enhancing vaccine efficacy and offering new strategies for tuberculosis prevention.

In the review series, eight topics are reviewed on advanced glycan decoding technologies, tumor immune response, acute myeloid leukemia (AML), aging and neurodegenerative diseases.

Advance analytical techniques are crucial for deciphering glycan codes, among which lectin microarrays and mass spectrometry are some of the most commonly used methods for analyzing glycosylation modifications. Lectin microarrays, leveraging the high specificity of lectins for glycan-binding, are suited for profiling the glycan spectra of diverse and complex biological samples. Yang *et al*. [5] review the use of lectin microarrays for profiling glycan spectra in various clinical samples. They emphasize advancements in lectin detection technologies and their applications in diagnosing and managing diseases such as tumors, autoimmune disorders, and chronic inflammation. This review provides a comprehensive overview of the potential of lectin microarrays in clinical glycobiology. Mass spectrometry, with its capability for both qualitative and quantitative analysis, has become the most commonly used analytical tool in glycomics and glycoproteomics research. Wang *et al*. [6] discuss recent advancements in N-glycan biomarker discovery for various human diseases. They highlight the analytical technologies that enable high-resolution glycomic analysis and the identification of disease-specific glycan signatures. This review demonstrates the clinical potential of N-glycans in disease diagnosis, prognosis, and therapeutic targeting. Bi and Tian [7] focus on the latest developments in mass spectrometry-based site- and structure-specific quantitative N-glycoproteomics. They explore the biomedical applications of N-glycosylation analysis, emphasizing its role in understanding disease mechanisms and identifying potential therapeutic targets.

Regarding the association of glycosylation with disease, a review by Cao *et al*. [8] summarize the multifaceted roles of glycosylation in the tumor immune response. They explore how aberrant glycosylation affects tumor development, progression, and immune evasion, and discuss recent advancements in glycan-based cancer immunotherapy. This review provides a theoretical framework for understanding glycosylation in tumor immunology and its therapeutic potential. Liu and Gu [9] review the post-translational modifications (PTMs) of FLT3, a receptor tyrosine kinase involved in acute myeloid leukemia (AML). They discuss how glycosylation and ubiquitination impact FLT3 function, stability, and subcellular localization, providing insights into potential therapeutic strategies for AML.

As the aging population continues to grow, it becomes a significant challenge for both economic and health systems. Aging is a complex process, which ultimately increases the risk of various diseases. Research suggests that protein glycosylation may play a key role in the mechanisms underlying the aging process and the pathogenesis of age-related diseases. A review by Zhang *et al*. [10] examines the association of protein glycosylation with aging and neurodegenerative diseases. They highlight the alterations in glycosylation patterns observed during aging and their implications in diseases such as Alzheimer’s and Parkinson’s. The studies included in this review cover different molecules from various sample sources. Another review by Wu *et al*. [11] focused on a widely-studied specific glycoprotein, IgG. They review the significance of IgG glycosylation in aging and aging-related diseases. They discuss how altered glycosylation patterns of IgG serve as biomarkers for these conditions and offer insights into disease mechanisms. This review highlights the potential of IgG glycosylation as a diagnostic and therapeutic tool in age-associated diseases.

Glycosylphosphatidylinositol (GPI) modifications further demonstrate the diverse forms in which glycosylation plays role within a biological system. Thus, this special issue also includes a review on GPI research. Li *et al*. [12] review the molecular and clinical aspects of inherited GPI deficiency. They outline the biosynthetic pathway of GPI-anchored proteins and summarize clinical cases of GPI deficiency, discussing current diagnostic and therapeutic approaches. This review provides a comprehensive understanding of GPI-related disorders and future research directions.

This special issue covers the role of glycosylation in various diseases, providing new insights and potential therapeutic strategies. The findings presented here highlight the critical importance of glycosylation research in advancing our understanding of disease mechanisms and improving clinical outcomes. Due to context limitations, many other important and interesting topics related to glycosylation could not be included. However, it is hoped that this special issue will showcase just the tip of the iceberg regarding the significance of glycosylation research, providing some reference for readers.
